# A Systematic Review of Industrial Exoskeletons for Injury Prevention: Efficacy Evaluation Metrics, Target Tasks, and Supported Body Postures

**DOI:** 10.3390/s22072714

**Published:** 2022-04-01

**Authors:** Ali Golabchi, Andrew Chao, Mahdi Tavakoli

**Affiliations:** 1 Department of Civil and Environmental Engineering, University of Alberta, Edmonton, AB T6G 1H9, Canada; alireza1@ualberta.ca; 2 Department of Electrical and Computer Engineering, University of Alberta, Edmonton, AB T6G 1H9, Canada; cusan@ualberta.ca

**Keywords:** exoskeletons, exosuits, wearable robots, wearable technologies, industrial exoskeletons, musculoskeletal disorders, injury prevention, systematic review

## Abstract

Industrial workplaces expose workers to a high risk of injuries such as Work-related Musculoskeletal Disorders (WMSDs). Exoskeletons are wearable robotic technologies that can be used to reduce the loads exerted on the body’s joints and reduce the occurrence of WMSDs. However, current studies show that the deployment of industrial exoskeletons is still limited, and widespread adoption depends on different factors, including efficacy evaluation metrics, target tasks, and supported body postures. Given that exoskeletons are not yet adopted to their full potential, we propose a review based on these three evaluation dimensions that guides researchers and practitioners in properly evaluating and selecting exoskeletons and using them effectively in workplaces. Specifically, evaluating an exoskeleton needs to incorporate: (1) efficacy evaluation metrics based on both subjective (e.g., user perception) and objective (e.g., physiological measurements from sensors) measures, (2) target tasks (e.g., manual material handling and the use of tools), and (3) the body postures adopted (e.g., squatting and stooping). This framework is meant to guide the implementation and assessment of exoskeletons and provide recommendations addressing potential challenges in the adoption of industrial exoskeletons. The ultimate goal is to use the framework to enhance the acceptance and adoption of exoskeletons and to minimize future WMSDs in industrial workplaces.

## 1. Introduction

Work-related Musculoskeletal Disorders (WMSDs) represent the leading type of occupational injuries in many countries. The US Bureau of Labor Statistics reported that WMSDs contributed to 26.1% of workplace incidents, which represented 266,530 days away from work for cases in 2019 [1]. Similarly, the economic burden of WMSDs in Canada is estimated to be 22 billion dollars annually [2]. With the introduction of exoskeletons to industrial workplaces, there has been a rising interest in the adoption of exoskeletons to reduce exposure to WMSDs and increase productivity [3,4].

The American Society for Testing and Materials (ASTM) defines an exoskeleton as “a wearable device that augments, enables, assists, and/or enhances physical activity through mechanical interaction with the body [5]”. The applications of exoskeletons are diverse; as body-worn devices, they can support a worker’s body and prevent injuries and improve performance by reducing physical demands. Although exoskeletons are being developed and used increasingly for industrial applications, the technology was previously adopted mostly for military and rehabilitation purposes [6]. It is expected that the total value of the exoskeleton market will reach $1.8 billion in 2025, an increase from $68 million in 2014 [7], which implies a high growth in the adoption of exoskeletons throughout different industries.

Although different industries have started exploring the adoption of exoskeletons as part of their operations, and some have already integrated exoskeletons into their workplace [8], the wide-scale adoption of industrial exoskeletons is still limited due to the unique challenges involved, especially related to evaluating their effectiveness for different applications. Although different studies have investigated the suitability of industrial exoskeletons using a variety of experiments and measurements, there is still limited information available regarding the impact of exoskeletons on different factors such as safety, productivity, and comfort, especially in the long term.

While several systematic reviews have been conducted in regard to the impacts of industrial exoskeletons, most studies have mainly focused on evaluation metrics (e.g., EMG, user satisfaction, and discomfort) to assess the effectiveness of a specific exoskeleton. However, it is important to also incorporate other parameters that can significantly impact the findings. In particular, the body postures adopted and the target tasks should be incorporated into the analysis in addition to the efficacy evaluation metrics. Therefore, the aim of this paper is to provide a systematic review of previous studies that have evaluated the effectiveness of industrial exoskeletons from the perspective of evaluation metrics, supported body postures, and target tasks.

## 2. Methods

The systematic review is implemented according to the Preferred Reporting Items for Systematic Reviews and Meta-Analyses guidelines (PRISMA) [9].

### 2.1. Literature Search

Search criteria were set up to identify published literature that evaluated passive exoskeletons for industrial applications. Different keywords used synonymously with exoskeletons (i.e., exosuits and wearable robots) were included in the search, and the search included exoskeletons developed to support different body parts and was not limited to a specific body part. Furthermore, keywords such as “occupational”, “work”, and “industrial” were used to highlight studies that have focused on exoskeletons that are developed for occupational applications. The defined keywords were used to search the databases using Boolean “AND” and “OR” operators. Filters were also applied to restrict the findings to those that were published between 1990 and 2021 and in English. The search criteria are summarized in Table 1.

### 2.2. Eligibility Criteria

In July 2021, the Scopus and PubMed online databases were searched to implement the systematic review. The search method described above resulted in 2561 initial studies. The studies were first filtered to remove duplicates based on their unique Digital Object Identifiers (DOIs). There were 255 duplicates found in the two databases. The remaining 2306 studies were then screened and filtered by applying the exclusion criteria to limit the studies to passive and industrial exoskeletons. Table 2 shows the exclusion criteria.

**Figure 1:**
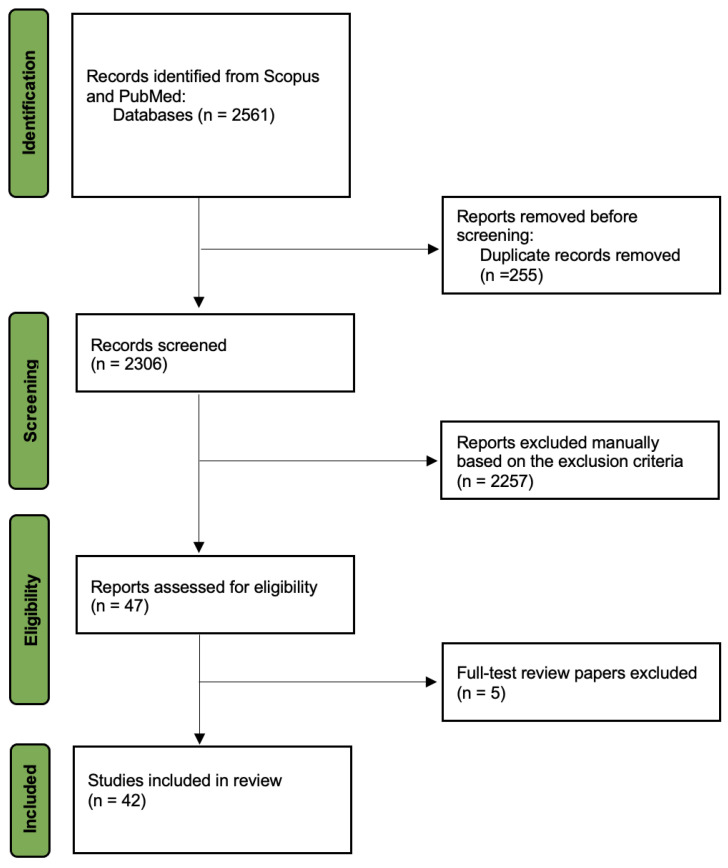
PRISMA flowchart of the systematic review (adopted from [9]).

The 2306 studies were manually screened based on their titles, abstracts, and keywords using the exclusion criteria. This process resulted in 47 studies. Among the 47 identified studies, 5 studies were systematic review papers and hence were removed. Therefore, 42 studies were identified for the systematic review. The PRISMA flowchart shown in Figure 1 demonstrates the systematic review process adopted. These 42 identified studies focused on the evaluation of industrial exoskeletons through experimentation and the use of evaluation metrics. The 42 studies were reviewed and analyzed to highlight and compare their evaluation metrics.

### 2.3. Data Analysis

The identified studies were thoroughly reviewed to identify the experiment setup, the evaluation features, and the experimental findings. The experiment setup includes the type of exoskeleton, the variables of the study, the demographics of the participants, and the experiment design. Evaluation features include the evaluation metrics (objective and subjective), the supported body postures, and the target tasks. Experimental findings include the findings of the studies and the benefits and/or drawbacks of the proposed methods.

## 3. Results

All studies in the review adopted at least one of the three evaluation features (i.e., evaluation metrics, body postures, and target tasks) to assess exoskeletons. The reviewed studies, along with their study method, evaluation approach, and the findings are shown in Table 3.

### 3.1. Exoskeleton Types

From the 42 studies identified, 40 assessed commercial exoskeletons. The brand, name, purpose, and number of papers that evaluated each exoskeleton are shown in Table 4. SuitX and Laveo were the most evaluated brands, with 12 studies evaluating Laveo exoskeletons and 10 evaluating SuitX. In addition, the exoskeleton that was evaluated the most was Laveo’s back support (12 studies). Out of the 42 studies, four studies either designed their own exoskeleton or did not mention the name of the exoskeleton evaluated.

### 3.2. Efficacy Evaluation Metrics

Evaluation metrics are categorized as objective and subjective metrics. Objective metrics are measured using experimental equipment (e.g., surface electrodes and motion sensors). Subjective metrics reflect a user’s perception and feedback in regard to the exoskeleton. Table 5 summarizes the evaluation metrics typically adopted to evaluate exoskeletons.

Out of the 42 studies in the systematic review, 26 used some form of subjective response, mainly including RPE and discomfort surveys. In terms of objective metrics, 33 studies used EMGS, 18 used motion capture, 8 used force plates, 8 evaluated heart rates, 7 evaluated the oxygen consumption and metabolic cost, 3 evaluated performance, 1 evaluated the range of motion, 1 evaluated hand grip to measure fatigue, and 1 evaluated the vibration of the shoulders. 

It is important to note that focusing only on efficacy evaluation metrics might not result in an inclusive analysis; as a result, similar studies can result in different findings in terms of the outcomes of the experiments. For example, Baltrusch et al. [48] used a variety of evaluation metrics such as EMG, motion capture, subjective responses, and oxygen consumption, and reported that the Laevo exoskeleton has a generally positive usability rating. In addition, Madinei et al. [30] used a similar methodology to Baltrusch et al. [48] and reported that using the Laveo exoskeleton made lifting and bending tasks easier and more efficient. However, Luger et al. [21] reported low wearability for the Laevo exoskeleton and Bosch et al. [51], using similar metrics, reported that Laveo led to discomfort in the chest region for static tasks. When evaluating the ShoulderX, a shoulder-supported exoskeleton, Van Engelhoven et al. [35] used EMG measurements and reported that the participants’ shoulder flexor muscle activity was reduced by up to 80%. However, De Bock et al. [42] reported that participants provided high discomfort scores in the shoulder region, and the usability was moderate. Thus, focusing only on efficacy evaluation metrics and not considering other evaluation features cannot provide a comprehensive analysis of the effectiveness of an exoskeleton.

### 3.3. Body Posture

The body posture feature reflects the required body position of the participants when performing the experiment tasks. The body posture adopted during the experiments is an important feature because it has a direct relationship with the impact of the exoskeleton on different body parts [52]. The most common body postures in the reviewed studies include pushing, pulling, twisting, sitting, standing, kneeling, bending, and squatting. Similar to efficacy evaluation metrics, the impact of different postures has to be investigated in conjunction with other evaluation features. Otherwise, the outcomes of the analysis might not properly reflect the suitability of the exoskeleton for different activities; studies that do not consider posture or that focus only on one posture can provide only limited information about the effectiveness of an exoskeleton.

For example, Wei et al. [50] studied lifting using the stoop posture and reported 35–61% lower muscle activity and a 22% lower metabolic cost when using the Mebot-EXO. Bosch et al. [51] also studied lifting using the stoop posture and indicated 35–38% lower back muscle activity and lower discomfort in the low back when using the Leavo exoskeleton. Although the findings of such studies provide valuable information about the impact of an exoskeleton on a specific posture, they lack further information about the comparison of different lifting postures and ignore the impact of the task on the selected posture and the effectiveness of the exoskeleton. Furthermore, Simon et al. [13] and Frost et al. [14] compared stoop, squat, and freestyle postures using EMG and motion capture data with VT-Lowe’s Exosuit and the PLAD exoskeleton, respectively. Simon et al. [13] reported that the results obtained from EMG and motion capture measurements for freestyle posture style were not significantly different from those for the squat posture style. Frost et al. [14] compared the same postures with the PLAD exoskeleton and showed that there was a significant reduction in erector spinae and L4/L5 flexion. While these studies provide more information on the role of different postures on the effectiveness of exoskeletons, incorporating further evaluation metrics as well as target tasks into the analysis can improve the applicability and generalizability of the findings.

### 3.4. Target Tasks

The target task evaluation feature represents the activity that the exoskeleton is used for. This feature is considered an important variable because defining the task enables evaluating the different postures and techniques that can be adopted to complete the task. All 42 studies evaluated at least one independent task. Out of the reviewed studies, 18 adopted manual handling tasks, 8 evaluated static tasks, and 17 selected tasks that required using tools (e.g., screwing, clip fitting, and drilling). Furthermore, 5 studies included tasks that required the participant to walk, 2 studies required the participant to climb, and 2 studies asked participants to perform experiments that involve balance (e.g., unipedal vs. bipedal stance). However, even when the same tasks are evaluated, the findings can vary due to other features such as the posture used to complete the task. Furthermore, the results of the analysis might differ when evaluating the same posture but for different tasks. For example, when evaluating a stoop posture, it is critical whether the task consists of dynamic stooping or squat lifting, as it impacts the results of the analysis.

### 3.5. Integration of Evaluation Features

Table 6 summarizes the evaluation metrics, postures, and tasks that each of the 42 reviewed studies adopted. Although most studies did not design experiments specifically to evaluate various tasks and postures using evaluation metrics, any experiment intending to assess the impact of exoskeletons requires, at a minimum, defining the task to be carried, either using a freestyle posture or a predetermined posture.

To properly evaluate exoskeletons, it is critical to incorporate all three dimensions into the analysis: efficacy evaluation metrics, supported body postures, and target tasks. If all dimensions are not properly incorporated, the impact of one feature (e.g., posture) on another (e.g., muscle activity) cannot be established thoroughly. For example, Baltrusch et al. [47] considered all three dimensions: evaluation metrics (muscle activity and metabolic consumption), supported body postures (upright postures), and target tasks (lifting a box) in their experiments, and reported that the metabolic consumption was higher in squatting compared to stooping. Furthermore, the authors reported that the participants felt more discomfort when carrying out the task in a squat posture versus a stooping posture. On the other hand, another study [48] used only two dimensions: evaluation metrics (subjective response and metabolic consumption) and target tasks (lifting a box). While this study specified a bending angle (between 0–20 degrees or greater than 20 degrees) in the lifting task, it did not specify the participants’ lifting postures. As a result, the findings only implied a decrease in metabolic costs when using the exoskeleton.

**Figure 2:**
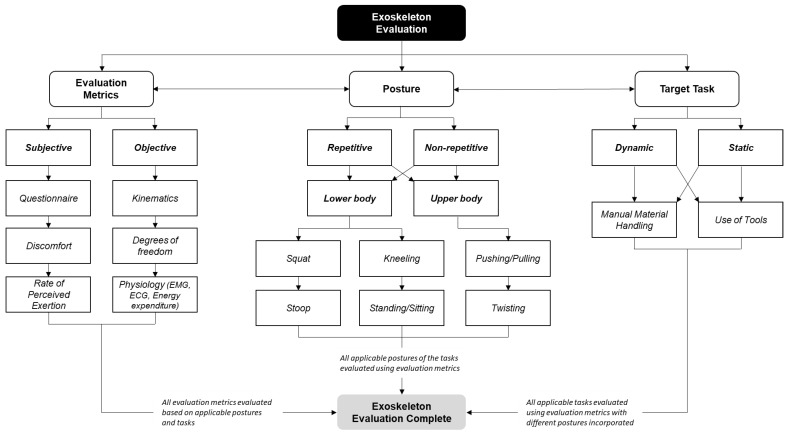
Framework for exoskeleton evaluation.

The review of previous studies indicates the importance of incorporating all three evaluation dimensions, including evaluation metrics, body posture, and target task when assessing exoskeletons to enable a practical and accurate analysis. The framework shown in Figure 2 is proposed to guide the proper evaluation of exoskeletons based on the three dimensions discussed. The proposed framework outlines the three evaluation dimensions that need to be investigated simultaneously. Efficacy evaluation metrics include both subjective and objective measurements, which are commonly considered in most of the previous studies. Subjective evaluations reflect participant responses (e.g., RPE, discomfort, and effectiveness) while carrying out a task with and without the exoskeleton. Objective evaluations include physiology (e.g., EMG) and kinematics (e.g., motion capture systems) and use measurements typically obtained through sensors to provide objective data. In addition to efficacy evaluation metrics, the different postures that can be adopted must be considered as part of experiment design, including repetitive and non-repetitive motions. In addition, the target task, reflecting the specific task and its dynamic or static nature (e.g., stationary standing vs. walking) needs to be incorporated into the experiment design, data collection, and analysis.

The three-dimensional iterative approach provides a thorough analysis of the physical, physiological, and postural impacts of using an exoskeleton. While this approach is more desirable for the evaluation of exoskeletons because it covers multiple aspects, it can also be more time-consuming and costly as compared to evaluation based on one or two dimensions. The intended outcome of the study is an important factor when deciding on which features to evaluate. For example, many of the reviewed studies incorporated two dimensions (e.g., EMG and a manual handling task) and were mostly interested in assessing a specific result (e.g., muscle activity). While these studies provide valuable insight on a specific outcome, they lack the comprehensiveness to provide findings that can guide the long-term implementation of the exoskeletons, especially for industrial adoption. As a result, a practical approach is to start the evaluation with one or two dimensions and add more features throughout the experiments to reflect on all three dimensions as more data are collected.

## 4. Conclusions

This study presented a systematic review of previous studies evaluating industrial exoskeletons. The reviewed studies adopted various evaluation features and reported findings dependent on different factors such as the exoskeleton features, the evaluation metrics, the posture used, and the task evaluated. The findings of the review highlighted that the state-of-the-art exoskeleton evaluation methods often consider one or two evaluation dimensions independently without further cross-validation. As the assessment of exoskeletons requires the integration of various factors, an evaluation framework is proposed that suggests a three-dimensional iterative evaluation approach to evaluate and adopt exoskeletons for industrial use.

## Figures and Tables

**Table 1:** Search criteria for the systematic review.

Operator	Criteria	Value
OR	Keywords	Exoskeleton exosuit wearable robot
OR	Keywords	Occupational work industrial
AND	Year	1990 and 2021
AND	Language	English

**Table 2:** Exclusion criteria for literature review.

Excluded Keywords		
Active/semi-passive exoskeletons	Military	Controlled-based exoskeletons
Rehabilitation	Enhancement of medical/surgical experience	Neuroprosthesis
Physical therapy	Virtual reality-based evaluation	Simulation modelling based evaluation

**Table 3:** Findings of reviewed studies on evaluation of exoskeletons.

Study	Exoskeleton	Study Method	Evaluation	Findings
[10]	Used their own Device	Participants: 9 healthy males (age: 23.9 ± 4.58 years, weight: 83 ± 10.99 kg, height: 1.84 ± 0.067 m) Procedure: Gathered Max Voluntary Static Contractions Lifted a wooden container with 3 different loads (5 kg, 15 kg, 25 kg) Started in anatomical position, picked up the box from the floor and placed it on a shelf	Measurements: Objective: EMG, percentage of Max Voluntary Static Contractions Subjective: Discomfort, perception of force and loss of movement Independent Variables: Load (5, 15, and 25 kg) Technique (Freestyle, Stoop, Squat) Suit vs. No suit Dependent variables: EMG for four muscles: TES, LES, RA, and EO Peak pelvis sagittal angle, peak lumbar angle, trunk, load vertical accelerations	↑ Loads on LES muscle activity and variance between participants ↓ Lumbar erector spinae activity ↓ Average percent 14.4% (SD 4.5%) for LES and 27.6% (SD 8.6%) for TES Usability 50% reported discomfort around the knees 20% replied ‘No’, 30% replied ‘yes’, and 50% replied ‘maybe’ for thicker knee pads
[11]	VT-Lowe’s exoskeleton	Participants: 12 young healthy males (age: 22.75 ± 4.35 years, weight: 80.41 ± 5.59 kg, height: 178.92 ± 6.05 cm, BMI: 25.16 ± 1.91 kg/m^2^) Procedure: Trained for 30 min Gathered MVC Lifted a box from the ground to neutral standing position, then put it back down Completed lifts with all combinations of variables in a random order	Measurements: EMG Independent Variables: Load: 0% and 20% of body weight With and without suit Freestyle, Squat, Stoop, Asymmetric Dependent Variables: Normalized averaged peak muscle activity for all muscles Normalized averaged mean muscle activity for all muscles)	↓ EMG for squat (peak: 35.4%, mean: 31.4%) ↓ Freestyle (peak: 32.3%, mean: 30.5%) ↓ Stoop lifting (peak: 27%, mean: 25.9%). Symmetric lifts had a higher peak EMG reduction for leg muscles on average
[12]	SPEXOR	Participants: 10 Healthy males (age: 56 ± 8.7 years, weight: 83.6 ± 16.2 kg, height: 1.75 ± 0.07 m) Procedure: Held a stoop for 5 s at 6 heights, 100% (upright), 95%, 80%, 60%, 20% and 0% (touching the floor) Lifted a 10 kg box with handles 10 cm above ankles to neutral standing, then placed it back down	Measurements: EMG Custom-made 1.0 × 1.0 m force plate to measure ground reaction forces at 200 Hz Opto-electronic 3D movement registration system; kinematics of the right side of the body were collected at a sample rate of 50 Hz Dependent Variables: Suit vs. no suit and squat, stoop, and freestyle techniques	↓ L5-S1 compression forces Lifting: ↓ Peak L5-S1 compression forces by 972 ± 216 N (14 ± 3%) The moment support at this instant was 33.4 ± 1.1 Nm compared to 40.8 ± 1.1 Nm maximally ↓ Peak trunk angular velocity 33 ± 9°/s (17 ± 5%) Peak compression forces were larger for squat than stoop
[13]	VT-Lowe’s Exosuit	Participants: 12 young men (age: 23.5 ± 4.42 years, height: 179.33 ± 6.37 cm, weight: 80.4 ± 5.59 kg) Procedure: Lifted a box from a 10 cm tall table to standing, then put it back down, finally back to standing. Task was repeated 4 times in a minute There were 12 trials; randomized order between participants Instructions for squat were to keep back straight; instructions for stoop were straight legs	Measurements: 120 Hz 8 camera motion capture Additional heights and angles were calculated in MATLAB using marker position data Independent Variables: With suit and without suit Lift style (Freestyle, Squat, and Stoop) Box weight, 0% and 20% of bodyweight Bending Down or lifting up; used for analyzing speed and acceleration Dependent Variables: Ankle and knee angles Angle between shoulder, hip, knee Shoulder elbow and wrist heights Lifting speed and acceleration	↑ 1.5 degree in ankle dorsiflexion ↓ 2.6 degree in knee flexion ↓ 2.3 degrees in SHK angle
[14]	PLAD	Participants: 13 men (age: 20.9 ± 3.8 years, height: 1.84 ± 0.05 m, weight: 82.0 ± 9.2 kg) Procedure: Gathered resting and MVC measurements Lifted a 15 kg box (0.37 × 0.33 × 0.27 m^3^)	Measurements: EMG 3D Electromagnetic Sensors Strain gauges Independent variables: Three lifting styles: stoop, squat, freestyle Six different PLAD tensions/elastic elements (approximate stiffness coefficients of 0 (no-PLAD), 300, 550, 800, 1050 and 1300 N/m) Dependent variables: Activity of latissimus dorsi, thoracic and lumbar erector spinae, rectus abdominis, external oblique, gluteus maximus, biceps femoris and rectus femoris	↓ Erector spinae activity (mean of thoracic and lumbar) in comparison to the no-PLAD condition for the stoop (37%), squat (38%), and freestyle (37%) lifts ↓ L4/L5 flexion moment for the stoop (19.0%), squat (18.4%), and freestyle (17.4%) lifts without changing peak lumbar flexion
[15]	Laevo V2.56	Participants: 39 males (age: 25.9 ± 4.6 years, weight: 73.5 ± 8.9 kg, height: 78.8 ± 7.3 cm, BMI: 22.9 ± 2.1 kg/m^2^, rest blood pressure of 129/79 ± 7.7 mmHg, 4 left-handed and 32 right-handed) Procedure: Two sets of five repetitions Picked up an 11.6-kg load (i.e., a 10-kg load placed in a 1.6-kg box (60 × 40 × 22 cm) with handles on both sides (19 cm) at approximately 70° trunk inclination (stoop)	Measurements: EMG Joint inclination angles measured using two-dimensional gravimetric position sensors Heart rate Independent variables: Techniques (squat, stoop) Orientations (frontal/symmetric, lateral/asymmetric) Exoskeleton (with, without) Dependent Variables: Trunk and hip extensor muscle activity (primary outcomes), abdominal, leg, and shoulder muscle activity, joint kinematics, and heart rate	↓ Median/peak activity of the erector spinae (≤6%) ↓ Biceps femoris (≤28%) ↓ Rectus abdominis (≤6%) ↑ Median/peak activity of the vastus lateralis (≤69%) ↑ Trapezius descendent (≤19%), and median knee (≤6%) ↑ Hip flexion angles (≤11%), ↓ Heart rate: 5 bpm (η2p = 0.40) ↑ Minimal, median, and maximal knee flexion by 3.0° (>100%), 4.9° (22.9%), ↑ maximal knee flexion by 2.2° (4.6%), ↑ 11.0% maximal hip flexion angle (6.7°) in a stoop lifting style
[16]	A new passive trunk exoskeleton system	Participants: 10 males (age: 33 ± 3 years, weight: 72 ± 3 kg, height: 172 ± 3 cm) with basic construction knowledge Procedure: Lifted a box onto a table from floor Carried the box to a destination	Measurements: EMG Subjective Independent variables: Load weight (5, 15, 25 kg) Posture (stoop vs. squat) With or without suit Dependent variables: Muscle activity Perceived discomfort Usability LPP test on shoulders, lower back and legs	↑ Muscle activity of TES, LES, RA, and EO with increasing lifting load Squat posture had higher LES sEMG activity than stoop posture with exosuit Stoop posture showed consistent higher LES sEMG activity than squat posture without exosuit For lifting posture, stoop posture had greater EO sEMG activity than squat ↓ LES muscle activity (11–33% MVC; max 32.71% MVC) ↓ Discomfort scores (42.40%) of the lower back at max load
[17]	BackX ACLaevo V2.5	Participants: 10 males (age: 25.2 ± 3.8 years, height: 176.4 ± 7.4 cm, and weight 76.7 ± 8.8 kg) and 10 females (age: 27.5 ± 2.7 years, height: 166.5 ± 5.4 cm, and weight: 61.2 ± 8.6 kg) Procedure: Gathered max voluntary Participants stood as still as possible, barefoot, arms crossed at chest and looking straight ahead for a minute	Measurements: 100 Hz Force platform Independent variables: Exosuit (BackX, Laevo, no suit) One foot vs. two Eyes open or closed Dependent variables: Center of pressure, mean frequency, and velocity	↑ COP median frequency and mean velocity during bipedal stance In unipedal stance, significant improvement in postural balance, especially among males, as indicated by smaller COP displacement and sway area, and a longer time to contact the stability boundary Larger effects of BSEs on postural balance were evident among males
[18]	FLx and V22 (strongArm Technologies)	Participants: 10 males (mean age: 24.9 ± 5.0 years (SD), range 22–38 years; weight: 81.1 ± 16.1 kg, range 63.4–102.7 kg; height: 179.4 ± 4.6 cm, range 172.1–186.4 cm) Procedure: Subjects had 10 min to become used to the suit Trained to use squat Subjects lifted a box to neutral standing position, then put it back down	Measurements: Body segment kinematics from motion capture system Force plates Independent variables: Main effects of intervention Lift origin height Lift origin asymmetry Load weight Suit (No suit, FLx, V22) Dependent variables: Kinematics Horizontal moment arms from the L5/S1 joint Three-dimensional spinal loads	↓ Peak torso flexion at the shin No differences in moment arms or spinal loads attributable to either of the interventions
[19]	Spexor	Participants: 7 males with minor back pain and 7 females with minor back pain (age: 40.5 ± 10.8 years; height: 174.5 ± 9.5 cm; weight: 76.6 ± 18.0 kg) Procedure: Used the test battery developed and used before by another study; included 12 tasks	Measurements: Subjective (scale from 1–10) Independent Variables: With and without exosuit Dependent Variables: Perceived task difficulty Discomfort (due to suit) Low back discomfort Objective performance based on task	The sit stand test was on average considered easier ↓ Lower low back discomfort scores
[20]	Skelex 360	Participants: 11 male trained plasterers Procedure: MVC was gathered Subjects plastered a room with 4 m^2^ walls and 2 m^2^ ceiling twice, one with suit the other without Plastering is separated into 3 steps: apply, screed, and finish	Measurements: EMG Subjective (RPE) Independent Variables: With exosuit or without suit Dependent Variables: Muscle activity in AD, MD, Trap, BB, TB, and PM Perceived exertion	↓ RPE for all activities except applying to wall ↓ EMG amplitudes of three agonist muscles (Trapezius and Medial Deltoid, and Biceps Brachii) ↓ EMG values in suit for most tasks
[21]	Laevo V2.56	Participants: 36 males (age: 25.9 (4.6) years, height: 178.8 (6.4) cm, weight: 73.5 (8.9) kg, BMI: 22.9 (2.1)) 4 left-handed; the rest were right-handed Procedure: Stair climbing test (7 stairs, up and down with no time limit) Stood up from a chair, walked 3 m, then back 3 m into the chair Picked and placed eight boxes (9.6 kg; 30 × 31 × 26 cm) with both hands from one pallet to another Fastened five screws in a metal bar using both hands in a forward bent position Picked and placed four boxes (5.9 kg; 20 × 30 × 34 cm) with both hands	Measurements: EMG 2D gravimetric position sensors Heart rate Subjective Independent Variables: With and without exosuit Side of the body to measure (randomly picked) Dependent Variables: Muscle activity in 6 muscles Performance Usability Comfort Heart rate Posture	Heart rate was not affected ↑ Task duration with exosuit ↑ Perceived task difficulty for stair climbing and TUG Wearer comfort was low and usability was good Supports hip extension by decreases of ~22% for lifting and ~20% for fastening The gastrocnemius medialis was tracked additionally and significantly increased during fastening and lattice box lifting (~21%) ↑ Knee and hip flexion during lifting tasks (27–36%), ↑ Knee extensor activity by ~20%
[22]	ShoulderX Mate Paexo	Participants: 2 males: right-handed automotive industry workers (age: 34 ± 3 years, weight: 87 ± 6 kg) Procedure: Gathered MVC Task was to tighten a M12 hex head cap screw with three different shoulder angles: above, below, and equal to 90 degrees	Measurements: EMG Frequency and amplitude Independent variables: Heavy vs. light tool Exosuit vs. no suit Dependent variables: Muscle activity in the shoulder Vibration	↓ Shoulder muscle activity for all three exoskeletons Minor differences in the vibrations acting on the different exoskeleton types Paexo exoskeleton seems to decrease shoulder muscle activity to a greater extent when compared to ShoulderX and Mate The impact of the weight of the tool was more than expected
[23]	SIAT lower limb exoskeleton with crutches	Participants: 3 males (age: 24.0 (1.0) years, weight: 64.8 (3.8) kg, height: 173.0 (2.0) cm) Procedure (fatigue experiment): Subjects worked out the arm muscles with a common piece of gym equipment Measured the subjects’ hand grip strength, asked them to fill out an RPE form Repeated 5 times Procedure (exosuit experiment): Walked across a room for 3 min wearing the suit	Measurements: EMG Hand grip (fatigue) Independent variables: The setting on the exosuit Dependent variables: Muscle activity Rate of fatigue	Strength remained almost constant in the first three sessions and decreased rapidly in the last two sessions ↑ Borg-RPE value In the exosuit experiment, the arms’ fatigue in Feedback was lower than the fatigue in NoFeedback The fatigue of two arms in BigStep was more unbalanced than that in NoFeedback
[24]	EksoVestprototype	Participants: 6 male participants (32.5 (11.8) yrs, 172.3 (4.6) cm, and 72.6 (9.1) kg) and 6 female participants (22.5 (1.5) yrs, 169.7 (5.2) cm, and 63.8 (6.2) kg) Procedure: Gathered MVC 2 tasks: overhead drilling and light assembly Participants were given a mock drill and told to put it into a hole without touching the sides and to maintain a certain level of force	Measurements: EMG Subjective Independent variables: Suit vs. no suit Overhead or shoulder height Weight of the drill (heavy vs. light) Dependent variables: Number of errors in drilling Muscle activity Speed of work	↓ Peak (up to ∼45%) and median muscle activity of several shoulder muscle groups (up to ∼50%) Wearing the suit made drilling almost 20% faster Wearing the suit made forearms more comfortable
[25]	EksoVest Prototype	Participants: 14 males and 13 females Procedure: Gathered the maximum voluntary range of motion for the shoulders Subjects stood on a force platform with eyes closed and feet together for 70 s Slip and trip risks were assessed by having participants walk across a track with two force platforms near the middle	Measurements: EMG Force platform Body kinematics (motion capture) Independent variables: Suit vs. no suit Dependent variables: Muscle activity Range of motion	↓ Maximum shoulder abduction ROM by ~10% ↑ Mean center of pressure velocity in the anteroposterior direction by ∼12% Vest use had minimal influence on trip-/slip-related fall risks during level walking ↓ Spine loadings (up to ∼30%) ↓ Peak AP shear (by 29.5%) and compressive forces (by 19.3%)
[26]	HeroWear Apex	Participants: 15 males and 5 females, 25.5 ± 4.7 years old (range 21–39), height: 178.5 ± 8.9 cm (range 167–192), weight: 79.7 ± 20.5 kg (range 51–144) All right-handed Procedure: Stood from a stool with two 7.9 kg dumbbells and lifted dumbbell from floor under dominant hand to standing Lifted plastic box with handles and 15-lb (6.8-kg) weight from floor in front of participant to waist level in sagittal plane using both arms and lowered same box from waist to floor Lifted 15 lb box from floor to elbow-high table 90 degrees to the right and walked across with 15lb box	Measurements: Kinematics EMG Heart rate Self-reported ratings Independent Variables: Suit engaged or not Different tasks Dependent Variables: Heart rate Muscle activity Posture Heart rate	↓ Mean EMG value with the engaged exosuit ~85% ↓ Peak ES EMG was similar to mean EMG ↓ Trunk flexion/extension ROM during asymmetric dumbbell lifting The engaged exosuit was mildly to moderately helpful Heart rate was not significantly affected
[27]	BackX and Laevo	Participants: 18 participants. Males: 25.3 (4.8) yrs, 74.0 (6.3) kg, and 175.9 (4.0) cm. Females: 24.0 (2.4) yrs, 64.9 (7.3) kg, and 165.6 (3.6) cm. Average 24.7 (3.7) yrs, 69.4 (8.2) kg, and 170.7 (6.5) cm Procedure: Participants were instructed to put pegs into 2 of 5 columns in a peg board as fast as they could Each participant completed all combinations of variables	Measurements: EMG Subjective Kinematics (motion capture) Independent variables: Suit (no suit, BackX, Laevo) Supported vs. unsupported (sitting) Work height (−20, 6, 48, 90 cm from floor) Work distance (0, 20, 30 cm from feet) Work orientation (0, 45, 90 degrees to the right) Dependent variables: Working posture Activity in secondary muscle groups Perceived balance Usability and comfort	↓ Lumbar flexion changes of <~140 Caused no significant changes in secondary muscles Extreme postures cause greater discomfort wearing the suit Many discrepancies between suits, tasks, genders, and individuals
[28]	PAEXO	Participants: 12 participants (24 ± 3 y, height: 176 ± 15 cm, weight: 73 ± 15 kg) Procedure: Screwing and drilling at about eye level 5 min duration	Measurements: EMG Oxygen consumption Heart rate Motion capture Independent variables: Suit vs. no suit Dependent variables: Muscle activity Heart rate Posture	↓EMG, heart rate, and oxygen rate
[29]	Laevo and BackX	Participants: 18 participants. Males: 26.8 (3.9) years, 178.4 (4.4) cm, 80.9 (5.0) kg. Females: 25.1 (3.1) years, 165.8 (4.3) cm, 62.5 (5.7) kg Procedure: 1 h of training with suit Lifted a box 10% of their body weight for 4 min 10 times lifting and lowering a minute	Measurements: Subjective EMG Motion Capture Energy expenditure Independent variables: Suit (backX vs. laevo vs. no suit) Height (mid shank and knee level) Symmetry (90 degrees to the right, but not from mid shank) Dependent variables: Perceived exertion Muscle activity Posture Oxygen consumption	↓ Peak levels of trunk extensor muscle activity (by ~9–20%) ↓ Reduced energy expenditure (by ~8–14%) Minimal changes in lifting behaviors using either BSE Use of both BSEs led to generally positive usability ratings Almost equal people preferred each exosuit
[30]	BackX and Laevo	Participants: 18 participants. Males: age 25.3 (4.8) years, weight 74.0 (6.3) kg, and height 175.9 (4.0) cm. Females: age 24.0 (2.4) years, weight 64.9 (7.3) kg, and height 165.6 (3.6) cm Procedure: Put pegs into 2 of 5 columns in a peg board as fast as they could Each participant completed all combinations of variables	Measurements: EMG Subjective Kinematics (motion capture) Independent variables: Suit (no exo, BackX, Laevo) Supported vs. unsupported (sitting) Work height (−20, 6, 48, 90 cm from floor) Work distance (0, 20, 30 cm from feet) Work orientation (0, 45, 90 degrees to the right) Dependent variables: Muscle activity Discomfort Posture	The beneficial effects appeared task- and gender-specific ↓ All three nEMG metrics in all of the six supported conditions using BackX ↓ Only two of the conditions using Laevo In the unsupported scenario, females reported lower RPEs when using either suit overall In the supported scenario, using a suit led to increased low-back RPEs for males Using suits had minimal effect on performance
[31]	PULE	Participants: 15 right-handed males (age of 28.6 ± 4.2 years old, weight of 68.5 ± 12.3 kg, height of 1.73 ± 0.15 m) Procedure: Participants held a wrench to a bolt overhead The first test had 50% rest for 50% wrench holding	Measurements: EMG Subjective Independent variables: Suit or no suit Work height (low, middle, high) Dependent variables: Muscle activity (AD, MD, TR, and TB) Rate of perceived discomfort (necks, shoulders, upper arms, forearms, upper backs, waists, and legs)	~20% of the participants reported discomfort, excessive force, or loss of range of motion at the arms The PULE was more effective when the bolt was higher ↓ Median nEMG values for the RAD, RMD, RTB, LAD, and LMD muscles and fatigue using the PULE system ↓ RPDs for shoulders, upper arms, and forearms wearing the PULE
[32]	Fawcett Exovest (arm), EksoWorks (shoulder), FORTIS (full)	Participants: 12 participants: 5 female, 7 male. Female mean age, body mass, and stature: 20.0 (1.1) years, 63.9 (8.7) kg, and 168.9 (6.1) cm. Male mean age, body mass, and stature: 22 (6.4) years, 71.4 (7.8) kg, and 174.9 (7.9) cm Procedure: First gathered MVC The task was overhead simulated drilling. The drill was inserted into a hole above the participant, and if the pressure fluctuated too much or the drill touched the walls it counted as a mistake	Measurements: EMG Subjective Performance Independent variables: Exosuit (arm, shoulder, full, no suit) Precision (Low (±5°), Middle (±3.5°), and High (±2°)) Dependent variables: Muscle activity RPE Number of errors	Higher precision demands increased some muscle activation levels and deteriorated quality Designs with supernumerary arms led to the largest reductions in quality and increased physical demands overall in the low back ↓ Shoulder demands ↓ Quality with the highest precision requirement
[33]	BackX, Laevo	Participants: 18 participants. Male age, stature, weight, and BMI: 24.4 (4.5) years, 176.5 (5.5) cm, 78.5 (7.0) kg, and 25.2 (2.7) kg/m^2^. Female age, stature, weight, and BMI: 25.1 (3.8) years, 167.4 (3.5) cm, 67.6 (9.4) kg, and 24.1 (3.4) kg/m^2^ Procedure: Two-hour training session MVC was gathered before trials Testing was made to replicate the lifting of a large object by lifting a 1.55 × 2.13 m wooden panel with handles (mass = 6.8 kg) Participants lifted for 5 min at 5 lifts per minute	Measurements: EMG Energy expenditure (portable indirect calorimeter) Subjective Independent variables: Posture (kneeling vs. standing) Symmetry (on the left or in front) Intervention (backX vs. laevo vs. no suit) Dependent variables: Muscle activity Energy expenditure Perceived discomfort Perceived balance Usability	↓ peak activity of the trunk extensor muscles (by ~10–28%) and energy expenditure (by ~4–13%) Subjective responses regarding perceived exertion and usability RPDs at the chest were higher in all conditions except symmetric kneeling At the waist, the Laevo led to significantly lower RPDs (1.5 [0.7]) compared to the SuitX (1.8 [1.1])
[34]	Levitate AIRFRAME	Participants: 11 male and 1 female automotive workers Half wore the suit; the other half did not Average age, weight, and height: 35 ± 5 years, 73.9 ± 4.9 kg, and 175.2 ± 5.3 cm Procedure: The workers wore the suits several times to work and became accustomed to them	Measurements: EMG Motion capture Independent variables: Suit vs. no suit Dependent variables: Muscle activity Posture	↓ Dangerous levels to 30% of the work time with the suit ↓ Deltoid (34%) and the trapezius (18%) muscular activities Referring to the posture, some differences were found in the range of movement of the back, neck, and arms owing to the use of the exoskeleton; however, the differences were smaller than 5% in all cases The trapezius never exceeded dangerous levels but the suit lowered muscle activity to even safer levels
[35]	ShoulderX	Participants: 13 males (age 37 ± 13 yrs, weight 81.2 ± 14.5 kg, and height 1.83 ± 0.08 m) All worked overhead 10 h a week Procedure: Gathered MVC Static test required participants to trace a line with a drill using a 90 degree shoulder flex Dynamic test required participants to lower their arms to pick up screws	Measurements: EMG Independent variables: Weight of drill (0.45 kg or 2.25 kg) Amount of support: no support, low support (8.5 Nm peak torque), medium support (13.0 Nm peak torque), and high support (20.0 Nm peak torque) Dependent variables: Muscle activity	↓ Wearer’s shoulder flexor muscle activity of UT, AD ↑ Strength of shoulderX by up to 80%. Subjects preferred the use of shoulderX over the unassisted condition for all task types
[36]	Skel-Ex	Participants: 5 males and 4 females All were workers experienced with making boats Procedure: Took place in the workplace Monitored workers under normal conditions, then monitored them wearing the suits	Measurements: Heart Rate Subjective Independent variables: Suit vs. no suit Dependent variables: Perceived exertion Cardiac cost Posture Rated usability	↓ Cardiac cost when wearing the PAD All the results for extreme and average indexes values are inferior when wearing the PAD Ratings were around 5/7
[37]	Chairless Chair	Participants: 46 healthy males (age: 24.8 ± 2.9 years, height: 182.6 ± 5.5 cm, weight: 78.1 ± 8.7 kg) Procedure: The experiment consisted of screwing, clip fitting, and cable mounting while standing	Measurements: Force platform EMG Motion capture Subjective Independent variables: Suit vs. no suit High or low setting on suit Working distances Dependent variables: Muscular activity Posture Perceived discomfort	↓ Physical load up to 64% of the subject’s body mass The COP remained with the lowest values of static postural stability for high sitting (27%) ↑ Vastus activity (∼95–135%) during sitting ↓ Gastrocnemius activity ~25%)
[38]	Crimson Dynamics, Skelex V1	Participants: 8 male automotive workers (age: 37.5 ± 13.0 years, height: 183.1 ± 3.4 cm, weight: 94.0 ± 8.6 kg, BMI: 28.1 ± 3.4 kg/m^2^) Procedure: The experiment took place at an automotive assembly workplace Workers wore a suit for a whole shift and were asked about their perceived exertion	Measurements: Subjective Independent variables: Intervention (suit 1 vs. suit 2 vs. no suit) Dependent variables: Perceived exertion	↓ Shoulders, anterior (right), shoulders, posterior, spine and whole-body using Crimson Dynamics’s device ↓ Elbow (right), neck, and spine for the Skelex exoskeleton
[39]	Ekso Vest, Ottobock Paexo, Comau Mate	Participants: 11 males, 6 females 8 worked at an automotive factory, 9 were students Mean age 25 (range 18–46) years, mean stature 174 (range 166–190) cm Procedure: The experiment included 3 tasks: twisting to pick up tools and screwing above the head and bending to pick up tools and screwing above the head	Measurements: ROM Motion capture Subjective Independent variables: Intervention (which suit or no suit) Dependent variables: Range of motion Posture Impression of suit	Paexo was the favorite for the subjects regarding ROM (12 subjects), followed by Ekso Vest (9 subjects) and Mate (which no subject selected as the best option) Four of the subjects chose both Paexo and Ekso Vest as the best option Paexo is the exoskeleton with smaller changes in body motion compared to Paexo and Ekso Vest
[40]	Paexo	Participants: 12 male college students (age: 23.2 ±1.2, height: 179.3 cm ±5.9 cm, and weight: 72.7 kg ±5.4 kg) 4 were left-handed Procedure: Used the right hand instead of the dominant hand, held a drill with their right and the top of the screen with their left The screen was overhead with a slight angle Moved a drill from a starting point to an end point and held it there for 2 s	Measurements: EMG Force plate Heart rate Oxygen consumption Motion capture camera Subjective Independent variables: With suit vs. no suit Dependent variables: Muscle activity Posture Oxygen consumption	↓ Shoulder physical strain and global physiological strain, without increasing low back strain nor degrading balance using Paexo These positive effects are achieved without degrading task performance
[41]	Prototype developed by IUVO	Participants: 18 male experienced automotive workers (age: 43.0 ± 11.1 yrs, height: 176.9 ± 5.5 cm, weight: 77.3 ± 9.1 kg) Procedure: Maintained a static posture: standing upright with extended arms while holding a 3.5 kg load The worker was requested to stop when feeling fatigue or discomfort Subjects traced a wavy line with arms almost extended, without lowering the arms until finished	Measurements: Subjective Independent variables: Suit vs. no suit Dependent variables: User acceptance Posture Performance RPE	Maintained the static posture for a mean time of 108.6 s with exosuit) and 157.8 s (without exosuit) with a 56% relative longer time length in the second case Score on the Borg scale was 3 (with exosuit) and 1.6 without exosuit) ↑ Endurance time during the dynamic task ↑ Precision and ↓ RPE when using the exosuit
[42]	ShoulderX, Skelex V2	Participants: 4 male industrial workers (age: 33.4 ± 5.7 years, weight: 80.9 ± 5.8 kg, height: 1.79 ± 0.02 m, worked for 9.3 ± 6.4 years) Procedure: MVC was gathered 6 common tasks were performed in the laboratory setting The suits were worn by workers doing their day-to-day activities	Measurements: Heart Rate EMG Subjective Independent variables: Suit (ShoulderX vs. Skelex vs. no suit) Dependent variables: Muscle activity Fatigue RPE	↓ Upper trapezius activity (up to 46%) and heart rate in isolated tasks ↓ Up to 26% upper trapezius activity reduction using both exoskeletons ShoulderX received high discomfort scores in the shoulder region and usability Skelex provide the most support during the in-field situations
[43]	Skelex MARK 1.3	Participants: 88 workers Procedure: 6 workstations where at least 30% of the work was overhead Subjects wore the suit for 30 min, slowly increasing duration until 2 h in a day	Measurements: Subjective Independent variables: Suit vs. no suit Dependent variables: Rating in questionnaire	↓ User acceptance and the intention of use
[44]	Chairless Chair	Participants: 45 males in experiment 1 8 participants in experiment 2 Procedure for experiment 1: On the first day, subjects sat in the Chairless Chair and performed an industrial task (screwing, clip fitting, and cable mounting) for about 20 min On the second day, subjects moved a dumbbell (3 kg) from a table on their right to a table on their left, and vice versa Procedure experiment 2: A rope was attached to the exosuit while the subjects sat, and slowly pulled them over	Measurements: Performance Force Independent variables 1: Position of the target object (3 levels) Setting of exosuit (3 settings) Independent variables 2: Setting of suit (5 settings) Dependent variables 1: Balance Dependent variables 2: Force required to induce a fall	Tilting moments of less than 30 nm were sufficient to let people fall backward when sitting on the exoskeleton Reaching for tools from different angles did not affect balance A further increase in postural control demands by any factor may significantly increase the risk of falling since the safety margin is lower when using the exoskeleton
[45]	EksoBionics’ EksoVest	Participants: 8 male assembly line workers Procedure: Subjects continuously moved nickel-sized stickers to different locations on a vertical structure (fixed metal ladder) between a range of 68–80 in from the floor	Measurements: Heart Rate Subjective Independent variables: Suit vs. no suit Dependent variables: Recovery time and Heart Rate Rest break frequency and RPE	↓ Average heart rate 3–18% in 65% of participants ↓ Heart rate range by 5–62% in 75% of participants 63% of participants had a faster recovery time Usefulness ratings were moderately favored
[46]	Spexor	Participants: 11 male luggage handlers (age: 47.4 ± 7.1 years, height: 175 ± 7 cm, and weight: 84 ± 15 kg) Procedure: Gathered MVC Lifted and lowered a box of 10 kg (0.39 × 0.37 × 0.11 m, with 2.5 cm diameter handles) from ankle height to hip height Lifting style was chosen by participant	Measurements: Oxygen consumption Force plate EMG Independent variables: Suit vs. no suit Dependent variables: Metabolic cost and muscle activity	↓ Net metabolic cost of lifting by 18% No significant effect on peak angles in knee flexion, hip flexion, lumbar flexion and trunk inclination No significant difference in positive and negative muscle work ↓ Back muscle activity
[47]	Laevo	Participants: 18 males Procedure: Participants completed a set of 12 tasks	Measurements: Subjective Independent variables: Suit vs. no suit Suit setting (low vs. high) Dependent variables: Energy expenditure Performance and RPE	↑ Objective performance in static forward bending ↓ Performance in tasks, such as walking, carrying, and ladder climbing Lifting and bending easier and more efficient, but harder on other tasks
[48]	Laevo	Participants: 13 males (age: 28.9 ± 4.4 years, height: 1.080 ± 0.04 m, weight: 76.9 ± 12.0 kg) Procedure: Two parts: walking and lifting First find preferred walking speed using the treadmill, then walk for 5 min Participants lifted and lowered a 10-kg box (0.39 × 0.37 × 0.11 m, with 2.5 cm diameter handles) at a rate of 6 lifts per minute	Measurements: Breathing gas analysis system EMG Kinematics (motion capture system) Independent variables: Suit vs. no suit Suit setting (high vs. low) Dependent variables: Metabolic cost and muscle activity	↓ Mechanical work generation ↑ Metabolic costs by 17% ↑ Abdominal muscle activity
[49]	Laevo	Participants: 5 males, 2 females as part of the questionnaire 2 males, 3 females as part of the EMG test Procedure: MVC gathered before Wore the suit at their normal industry jobs, starting with half an hour a day and ending with a full day wearing the suit Three tasks: Moved small pieces of wood off a conveyor onto a pallet Adjusted wooden slats to fit on a pallet Lifted a board to an inspection table, inspecting it, and moving it to another table	Measurements: Subjective EMG Independent variables: Suit vs. no suit Dependent variables: Borg CR-10, Scale, Likert Scale and a body map with a Visual-Analog Scale Muscle activity	↑ Overall effort and discomfort in the neck, shoulders, thoracic region, lumbar region and hips, and thighs ↓ Muscle activity between 0.8 and 3.8% of the back muscles
[50]	MeBot-EXO	Participants: 8 males (age: 24 ± 2.54 years old, height: 172.1 ± 5.89 cm, weight: 65.25 ± 6.98 kg) Procedure: Held a stoop posture for 5 min	Measurements: EMG Breath analysis Independent variables: Suit vs. no suit Dependent variables: Muscle activity and metabolic cost	↓ Muscle activity (by 35%~61%) in the static holding experiment ↓ Metabolic cost of energy (by 22%)
[51]	Laevo	Participants: 9 males and 9 females, mean age: 25 (±8) years, weight: 71 (±12.4) kg, height: 1.76 (±0.1) m Procedure: Participants manipulated pegs in a pegboard Participants held a stoop posture until they gave a rating of slight discomfort on the Borg scale	Measurements: EMG Subjective Motion capture system Independent variables: Suit vs. no suit Dependent variables: Muscle activity Discomfort Kinematics	↓ Muscle activity (by 35–38%) and lower discomfort in the low back in assembly task ↓ Hip extensor activity ↑ Discomfort in the chest region ↑ Endurance time from 3.2 to 9.7 min in the static holding task

**Table 4:** Exoskeletons evaluated in the identified studies.

Purpose	Exoskeleton	Number of Papers
Back support	BackX (SuitX), Laevo™ V2.5, SPEXOR, Apex	20
Shoulder support	ShoulderX (SuitX), SkelEx V1/V2 (SkelEX), Skelex 360 (Skelex)),CDYS (Crimson Dynamics), Mate (Comau), PAEXO (Ottobock), EksoVest (EksoBionics), AIRFRAMETM (Levitate), SPEXOR (SPEXOR)	18
Leg support	LegX (SuitX)	1
Standing/Sitting support	Chairless Chair (Noonee)	2

**Table 5:** Most common evaluation metrics adopted in evaluating exoskeletons.

Type	Metric	Measurement Device/Method	Purpose	Application for Exoskeleton Experiments
Objective	Electromyography (EMG)	Surface electrodes placed on skin	Record the electrical activity produced by skeletal muscles	Measure the magnitude of maximal voluntary isometric contraction (MVIC)
	Energy Expenditure	Indirect calorimetry	Measure the oxygen and carbon dioxide consumption	Determine the change in calories
	Electrocardiogram (ECG, EKG)	Surface electrodes placed on chest	Record the electrical activity produced by heart muscles	Determine the changes in heart rate
	Motion Capture	Motion sensors	Record the body movement during a physical activity	Determine the body kinematics
Subjective	Rate of Perceived Exertion (RPE)	Borg’s scale	Rate the perceived exertion after a defined physical activity	Determine the physical demands
	Discomfort Survey	Questionnaire	Measure body local discomfort	Determine the physical discomfort
	General feedback	Questionnaire	Record the user feedback and comments	Determine the usability and acceptance

**Table 6:** Exoskeletons evaluated in the identified studies.

Study	Evaluation Metric	Posture	Task
[10]	EMG; Subjective	Squat; Stoop; Freestyle	Manual handling
[11]	EMG	Squat; Stoop; Freestyle; Asymmetric	Manual handling
[12]	EMG; Force plate; Motion capture	Squat; Stoop; Freestyle	Manual handling
[13]	Motion Capture	Stoop; Squat; Freestyle	Manual Handling
[14]	EMG; Motion capture	Stoop; Squat; Freestyle	Manual handling
[15]	EMG; Motion capture; Heart rate	Stoop; Squat	Manual handling
[16]	EMG; Subjective	Stoop; Squat	Manual handling
[17]	Force platform (Center of Pressure)	-	Balance
[18]	Motion capture; Force platform	Squat	Manual handling
[19]	Subjective; Performance	Squat; Stoop	Walking; Climbing; Manual handling
[20]	EMG; Subjective	Overhead work	Use of tool
[21]	EMG; Motion capture; Heart rate; Subjective	-	Stairs; Manual handling; Static task
[22]	EMG; Vibration of shoulders	Overhead work	Use of tool
[23]	EMG; Hand Grip (fatigue)	-	Walking
[24]	EMG; Subjective	Overhead work	Use of tool
[25]	EMG; Force plate; Motion capture	Overhead work	Use of tool; Balance; Walking
[26]	EMG; Motion capture; Heart rate; Subjective	-	Manual handling
[27]	EMG; Motion capture; Subjective	-	Static task
[28]	EMG; Motion Capture; Heart rate; Oxygen consumption	Overhead work	Use of tool
[29]	EMG; Motion Capture; Subjective; Oxygen consumption	-	Manual handling
[30]	EMG; Motion capture; Subjective	-	Static task
[31]	EMG; Subjective	Overhead work	Use of tool
[32]	EMG; Subjective; Performance	Overhead work	Use of tool
[33]	EMG; Subjective; Oxygen consumption	Standing; Kneeling	Manual handling
[34]	EMG; Motion Capture	Overhead work	Use of tool
[35]	EMG	Overhead work	Use of tool
[36]	Subjective; Heart rate	Overhead work	Use of tool
[37]	EMG; Motion capture; Subjective; Force platform	-	Static tasks
[38]	Subjective	Overhead work	Use of tool
[39]	Motion capture; Subjective; Range of motion	Overhead work	Use of tool
[40]	EMG; Motion capture; Subjective; Heart rate; Force plate; Oxygen consumption	Overhead work	Use of tool
[41]	Subjective; Video review	Stoop	Manual handling; Static task
[42]	EMG; Subjective; Heart rate	Overhead work	Use of tool
[43]	Subjective	Overhead work	Use of tool
[44]	Performance; Force plate	-	Static tasks; Inducing falls
[45]	Subjective; Heart rate	Overhead work	Use of tool
[46]	EMG; Force Plate; Oxygen consumption	-	Manual handling
[47]	Subjective; Performance	Squat; Stoop	Walking; Climbing; Manual handling
[48]	EMG; Motion capture; Oxygen consumption	-	Manual handling; Walking
[49]	EMG; Subjective	-	Manual handling
[50]	EMG; Oxygen consumption	Stoop	Static task
[51]	EMG; Motion capture; Subjective	Stoop	Static task

## References

[1] Bureau of Labor Statistics (BLS) (2021). Survey of Occupational Injuries and Illnesses Data. https://www.bls.gov/iif/soii-data.htm#archive.

[2] Workers Health and Safety Center (WHSC) (2016). The Economics of Ergonomics. https://www.whsc.on.ca/Files/Resources/Ergonomic-Resources/RSI-Day-2016_MSD-Case-Study_The-economics-of-ergon.aspx.

[3] Bogue R. (2018). Exoskeletons—A Review of Industrial Applications. Ind. Robot Int. J..

[4] Hoffmann N., Prokop G., Weidner R. (2021). Methodologies for evaluating exoskeletons with industrial applications. Ergonomics.

[5] (2020). Standard Terminology for Exoskeletons and Exosuits.

[6] Pesenti M., Antonietti A., Gandolla M., Pedrocchi A. (2021). Towards a functional performance validation standard for industrial low-back exoskeletons: State of the art review. Sensors.

[7] ABI Research (2015). Exoskeleton Market to Reach $1.8B in 2025. https://www.abiresearch.com/press/abi-research-predicts-robotic-exoskeleton-market-e/.

[8] Marinov B. (2019). Toyota’s Woodstock Plant Makes the Levitate AIRFRAME Exoskeleton Mandatory Personal Protective Equipment. https://exoskeletonreport.com/2019/02/toyotas-woodstock-plant-makes-the-levitate-airframe-exoskeleton-mandatory-personal-protective-equipment/.

[9] Moher D., Liberati A., Tetzlaff J., Altman D.G., PRISMA Group (2009). Preferred reporting items for systematic reviews and meta-analyses: The PRISMA statement. Ann. Intern. Med..

[10] Abdoli M.-E., Agnew M.J., Stevenson J.M. (2006). An on-body personal lift augmentation device (PLAD) reduces EMG amplitude of erector spinae during lifting tasks. Clin. Biomech..

[11] Alemi M.M., Geissinger J., Simon A.A., Chang S.E., Asbeck A.T. (2019). A passive exoskeleton reduces peak and mean EMG during symmetric and asymmetric lifting. J. Electromyogr. Kinesiol..

[12] Koopman A.S., Näf M., Baltrusch S.J., Kingma I., Rodriguez-Guerrero C., Babič J., de Looze M.P., van Dieën J.H. (2020). Biomechanical evaluation of a new passive back support exoskeleton. J. Biomech..

[13] Simon A.A., Alemi M.M., Asbeck A.T. (2021). Kinematic effects of a passive lift assistive exoskeleton. J. Biomech..

[14] Frost D.M., Abdoli M.-E., Stevenson J.M. (2009). PLAD (personal lift assistive device) stiffness affects the lumbar flexion/extension moment and the posterior chain EMG during symmetrical lifting tasks. J. Electromyogr. Kinesiol..

[15] Luger T., Bär M., Seibt R., Rieger M.A., Steinhilber B. (2021). Using a back exoskeleton during industrial and functional tasks—Effects on muscle activity, posture, performance, usability, and wearer discomfort in a laboratory trial. Hum. Fact..

[16] Antwi-Afari M.F., Li H., Anwer S., Li D., Yu Y., Mi H.Y., Wuni I.Y. (2021). Assessment of a passive exoskeleton system on spinal biomechanics and subjective responses during manual repetitive handling tasks among construction workers. Saf. Sci..

[17] Park J.H., Kim S., Nussbaum M.A., Srinivasan D. (2021). Effects of two passive back-support exoskeletons on postural balance during quiet stance and functional limits of stability. J. Electromyogr. Kinesiol..

[18] Picchiotti M.T., Weston E.B., Knapik G.G., Dufour J.S., Marras W.S. (2019). Impact of two postural assist exoskeletons on biomechanical loading of the lumbar spine. Appl. Ergon..

[19] Kozinc Ž., Baltrusch S., Houdijk H., Šarabon N. (2021). Short-term effects of a passive spinal exoskeleton on functional performance, discomfort and user satisfaction in patients with low back pain. J. Occup. Rehabil..

[20] de Vries A.W., Krause F., de Looze M.P. (2021). The effectivity of a passive arm support exoskeleton in reducing muscle activation and perceived exertion during plastering activities. Ergonomics.

[21] Luger T., Bär M., Seibt R., Rimmele P., Rieger M.A., Steinhilber B. (2021). A passive back exoskeleton supporting symmetric and asymmetric lifting in stoop and squat posture reduces trunk and hip extensor muscle activity and adjusts body posture—A laboratory study. Appl. Ergon..

[22] Pinho J.P., Taira C., Parik-Americano P., Suplino L.O., Bartholomeu V.P., Hartmann V.N., Umemura G.S., Forner-Cordero A. A comparison between three commercially available exoskeletons in the automotive industry: An electromyographic pilot study. Proceedings of the 2020 8th IEEE RAS/EMBS International Conference for Biomedical Robotics and Biomechatronics (BioRob).

[23] Duan S., Wang C., Li Y., Zhang L., Yuan Y., Wu X. A Quantifiable Muscle Fatigue Method Based on sEMG during Dynamic Contractions for Lower Limb Exoskeleton. Proceedings of the 2020 IEEE International Conference on Real-Time Computing and Robotics (RCAR).

[24] Kim S., Nussbaum M.A., Esfahani M.I.M., Alemi M.M., Alabdulkarim S., Rashedi E. (2018). Assessing the influence of a passive, upper extremity exoskeletal vest for tasks requiring arm elevation: Part I—Expected effects on discomfort, shoulder muscle activity, and work task performance. Appl. Ergon..

[25] Kim S., Nussbaum M.A., Esfahani M.I.M., Alemi M.M., Jia B., Rashedi E. (2018). Assessing the influence of a passive, upper extremity exoskeletal vest for tasks requiring arm elevation: Part II—Unexpected effects on shoulder motion, balance, and spine loading. Appl. Ergon..

[26] Goršič M., Song Y., Dai B., Novak D. (2021). Evaluation of the HeroWear Apex back-assist exosuit during multiple brief tasks. J. Biomech..

[27] Kim S., Madinei S., Alemi M.M., Srinivasan D., Nussbaum M.A. (2020). Assessing the potential for “undesired” effects of passive back-support exoskeleton use during a simulated manual assembly task: Muscle activity, posture, balance, discomfort, and usability. Appl. Ergon..

[28] Schmalz T., Schändlinger J., Schuler M., Bornmann J., Schirrmeister B., Kannenberg A., Ernst M. (2019). Biomechanical and metabolic effectiveness of an industrial exoskeleton for overhead work. Int. J. Environ. Res. Public Health.

[29] Madinei S., Alemi M.M., Kim S., Srinivasan D., Nussbaum M.A. (2020). Biomechanical assessment of two back-support exoskeletons in symmetric and asymmetric repetitive lifting with moderate postural demands. Appl. Ergon..

[30] Madinei S., Alemi M.M., Kim S., Srinivasan D., Nussbaum M.A. (2020). Biomechanical evaluation of passive back-support exoskeletons in a precision manual assembly task: Expected effects on trunk muscle activity, perceived exertion, and task performance. Hum. Fact..

[31] Yin P., Yang L., Qu S., Wang C. (2020). Effects of a passive upper extremity exoskeleton for overhead tasks. J. Electromyogr. Kinesiol..

[32] Alabdulkarim S., Kim S., Nussbaum M.A. (2019). Effects of exoskeleton design and precision requirements on physical demands and quality in a simulated overhead drilling task. Appl. Ergon..

[33] Alemi M.M., Madinei S., Kim S., Srinivasan D., Nussbaum M.A. (2020). Effects of two passive back-support exoskeletons on muscle activity, energy expenditure, and subjective assessments during repetitive lifting. Hum. Fact..

[34] Iranzo S., Piedrabuena A., Iordanov D., Martinez-Iranzo U., Belda-Lois J.M. (2020). Ergonomics assessment of passive upper-limb exoskeletons in an automotive assembly plant. Appl. Ergon..

[35] Van Engelhoven L., Poon N., Kazerooni H., Barr A., Rempel D., Harris-Adamson C. (2018). Evaluation of an adjustable support shoulder exoskeleton on static and dynamic overhead tasks. Proceedings of the Human Factors and Ergonomics Society Annual Meeting.

[36] Moyon A., Poirson E., Petiot J.F. (2018). Experimental study of the physical impact of a passive exoskeleton on manual sanding operations. Proced. CIRP.

[37] Luger T., Seibt R., Cobb T.J., Rieger M.A., Steinhilber B. (2019). Influence of a passive lower-limb exoskeleton during simulated industrial work tasks on physical load, upper body posture, postural control and discomfort. Appl. Ergon..

[38] Hefferle M., Snell M., Kluth K. Influence of two industrial overhead exoskeletons on perceived strain—A field study in the automotive industry. Proceedings of the International Conference on Applied Human Factors and Ergonomics.

[39] Luque E.P., Högberg D., Pascual A.I., Lämkull D., Rivera F.G. Motion behavior and range of motion when using exoskeletons in manual assembly tasks. Proceedings of the SPS2020: Swedish Production Symposium.

[40] Maurice P., Čamernik J., Gorjan D., Schirrmeister B., Bornmann J., Tagliapietra L., Latella C., Pucci D., Fritzsche L., Ivaldi S. (2019). Objective and subjective effects of a passive exoskeleton on overhead work. IEEE Trans. Neural Syst. Rehab. Eng..

[41] Spada S., Ghibaudo L., Carnazzo C., Gastaldi L., Cavatorta M.P. Passive upper limb exoskeletons: An experimental campaign with workers. Proceedings of the Congress of the International Ergonomics Association.

[42] De Bock S., Ghillebert J., Govaerts R., Elprama S.A., Marusic U., Serrien B., Jacobs A., Geeroms J., Meeusen R., De Pauw K. (2020). Passive shoulder exoskeletons: More effective in the lab than in the field?. IEEE Trans. Neural Syst. Rehab. Eng..

[43] Ferreira G., Gaspar J., Fujão C., Nunes I.L. Piloting the use of an upper limb passive exoskeleton in automotive industry: Assessing user acceptance and intention of use. Proceedings of the International Conference on Applied Human Factors and Ergonomics.

[44] Steinhilber B., Seibt R., Rieger M.A., Luger T. (2020). Postural control when using an industrial lower limb exoskeleton: Impact of reaching for a working tool and external perturbation. Hum. Fact..

[45] Daratany C., Taveira A. Quasi-experimental study of exertion, recovery, and worker perceptions related to passive upper-body exoskeleton use during overhead, low force work. Proceedings of the International Conference on Human Interaction and Emerging Technologies.

[46] Baltrusch S.J., Van Dieën J.H., Koopman A.S., Näf M.B., Rodriguez-Guerrero C., Babič J., Houdijk H. (2020). SPEXOR passive spinal exoskeleton decreases metabolic cost during symmetric repetitive lifting. Eur. J. Appl. Physiol..

[47] Baltrusch S.J., van Dieën J.H., Bruijn S.M., Koopman A.S., van Bennekom C.A.M., Houdijk H. The effect of a passive trunk exoskeleton on functional performance and metabolic costs. Proceedings of the International Symposium on Wearable Robotics.

[48] Baltrusch S.J., Van Dieën J.H., Bruijn S.M., Koopman A.S., Van Bennekom C.A.M., Houdijk H. (2019). The effect of a passive trunk exoskeleton on metabolic costs during lifting and walking. Ergonomics.

[49] Cardoso A., Colim A., Sousa N. (2020). The Effects of a Passive Exoskeleton on Muscle Activity and Discomfort in Industrial Tasks. Occupational and Environmental Safety and Health II.

[50] Wei W., Wang W., Qu Z., Gu J., Lin X., Yue C. (2020). The effects of a passive exoskeleton on muscle activity and metabolic cost of energy. Adv. Robot..

[51] Bosch T., van Eck J., Knitel K., de Looze M. (2016). The effects of a passive exoskeleton on muscle activity, discomfort and endurance time in forward bending work. Appl. Ergon..

[52] Ebrahimi A. Stuttgart Exo-Jacket: An exoskeleton for industrial upper body applications. Proceedings of the 2017 10th International Conference on Human System Interactions (HSI).

